# Breast papillomas: current management with a focus on a new diagnostic and therapeutic modality

**DOI:** 10.1186/1477-7800-3-1

**Published:** 2006-01-17

**Authors:** W Al Sarakbi, D Worku, PF Escobar, K Mokbel

**Affiliations:** 1 The Breast Care Centre, St. George's & The Princess Grace Hospitals, London, UK; 2 The Cleveland Clinic Foundation, OH 44195, USA

## Abstract

Breast papilloma is a term that describes an intraductal papillary configuration of the mammary epithelium on macroscopic or microscopic examination. It includes solitary intraductal papillomas, multiple papillomas, papillomatosis, and juvenile papillomatosis (JP).

Recent advances in mammary ductoscopy (MD) have raised new possibilities in the diagnosis and treatment of breast papillomas. This technique represents an important diagnostic adjunct in patients with pathological nipple discharge (PND) by allowing direct visualisation and biopsy of intraductal lesions and guiding duct excision surgery.

Treatment of breast papillomas often entails surgical duct excision for symptomatic relief and histopathological examination. Recently, more conservative approach has been adapted. MD-assisted microdochectomy should be considered the procedure of choice for a papilloma-related single duct discharge. Furthermore, there is increasing evidence that MD has the potential to reduce the number of duct excision procedures and minimise the extent of surgical resection. Imaging-guided vacuum-assisted core biopsy can be diagnostic and therapeutic for papillomas seen on mammography and/or ultrasound.

Patients with multiple papillomas do have an increased risk of developing cancer and should be kept under annual review with regular mammography (preferably digital mammography) if treated conservatively. Magnetic resonance (MR) can be also used in surveillance in view of its high sensitivity. Because the risk is small, long term and affects both breasts, long-term follow-up is more appropriate than prophylactic mastectomy. Patients who prove to have solitary duct papilloma have insufficient increase in the risk of subsequent malignancy to justify routine follow-up.

## Introduction

Breast papillomas are a variety of lesions in the breast that are characterized by a papillary configuration on gross or microscopic examination (or both). Papillomas consist of a few broad fronds, abundant stroma, and an epithelium containing both luminal and myoepithelial cells [1]. These include solitary intraductal papillomas, multiple papillomas, papillomatosis, and juvenile papillomatosis (JP). Solitary intraductal papillomas are tumours of major lactiferous ducts, most frequently observed in women 30 to 50 years of age. They commonly cause a serous or sero-sanguinous discharge. The discharge is bloodstained in approximately one half of women with these papillomas [2]. Pathological nipple discharge (PND) is a relatively common symptom accounting for approximately 5% of all women attending symptomatic breast clinics [3]. Papilloma is the commonest pathological finding in women with PND accounting for 40% to 70% of cases [3,4], followed by adenomatous or papillary epithelial proliferations (14%). However the incidence of malignancy (invasive or in situ) as a cause of PND varies between 1 and 23% depending upon the series studied [3]. A solitary papilloma is not thought to be a pre-malignant lesion [5] and is considered by some to be an aberration rather than a true disease process [6].

Multiple intraductal papillomas occur in approximately 10% of cases of intraductal papillomas. Compared to solitary intraductal papillomas, multiple intraductal papillomas tend to occur in the younger patients, are less often associated with nipple discharge, more frequently peripheral, and more often bilateral. Essentially, these lesions appear to be susceptible to the development of carcinoma [1]. Carter [7] reported that 2 of 6 patients with multiple papillomata developed cancer, while only 4 of 58 with solitary papilloma developed cancer. Haagensen et al. [5] found that 5 of 51 patients with multiple papillomata developed cancer, which was a marked contrast to those with a solitary papilloma, where only 4 of 174 developed cancer. In Haagensen's series of 68 patients with multiple papillomas, simultaneous or subsequent carcinoma of the apocrine papillary and cribriform types was observed in 22 patients (32%) [8]. These findings were confirmed by another study, in which surgically excised specimens from patients with intraductal papillomas were subjected to three dimensional reconstruction [9]. All 16 cases of multiple papillomas in the series were found to originate in the most peripheral portion of the duct system; the terminal duct lobular unit. Furthermore, malignancy was associated with these multiple peripheral papillomas in six cases (37.5%). In contrast, no cases of carcinoma were found to be associated with solitary papillomas involving the large ducts. These findings suggest that peripheral papillomas, on the contrary to solitary central papillomas, may be highly susceptible to malignant transformation.

Papillomatosis is a term used to describe microscopic foci of intraductal hyperplasia that have papillary architecture and are, therefore included by Dupont and Page in the category of proliferative lesions without atypia [10]. JP is a rare condition that affects women between the ages of 10 and 44. Patients typically present with a painless mass that, on physical examination, is circumscribed, easily mobile, and most often thought to be a fibroadenoma [11]. Follow up studies have suggested that JP is associated with an increased risk of breast cancer in the patient's female relatives, and the patient herself may be at increased risk for developing carcinoma, particularly if the lesion is bilateral and the patient has a family history of breast cancer [11-14].

## Diagnostic methods

Intraductal papillary lesions of the breast continue to be one of the most difficult diagnostic and therapeutic problems for the surgeon as they may be histologically benign, borderline, or malignant. Subsequent surgical treatment depends largely on the histological appearance of the lesion.

### Physical examination

In patients with PND who do not exhibit palpable tumours, the localization of intraductal papilloma should be confirmed by localizing the orifice of the affected duct on the surface of the nipple and applying pressure with the fingertip to the circumference of the areola. The secretion of serous or bloody nipple discharge does not distinguish intraductal papilloma from cancer [15]. When a tumour is revealed by delicate palpation it should be characterized in terms of the number of tumours, size, consistency, surface and margin.

### Mammography

Simple mammography should be performed in all patients complaining of bloody or serous PND before ductography especially if they are aged 35 years or over [16]. Intraductal papilloma cannot be detected by conventional mammography. If micro-calcification (MCC) is identified on mammography, then further investigations in the form of stereotactic core biopsy are required especially if the MCC is polymorphic, clustered, or linearly distributed. Conventional analogue mammography, as a screening tool for breast cancer, has a positive predictive value of only 25%. The diagnostic yield is particularly low in young women who have dense breasts [17]. This diagnostic yeild can be improved by using digital mammogaphywhich has been shown to be significantly better than film mammography among women younger than 50 years compared with those who were at least 50 (P = 0.002). Moreover, digital mammography was more accurate than film mammography among women with heterogeneously dense or extremely dense breasts (P = 0.003) [18]. Furthermore, computer-aided detection (CAD) can further improve the sensitivity of digital mammography in detection MCC [19].

### Ductography

Ductography is a safe, simple technique for the visualization of the affected duct system in patients with PND. Intraductal papilloma may be demonstrated by filling defects within dilated ducts. Solitary papilloma is usually observed in the collecting ducts, while multiple papillomas are frequently observed in the branching ducts in a segmental or sub-segmental distribution leading in some cases to cystic dilatation of the ductal system. Distortion, narrowing or obstruction of the ducts may indicate the presence of malignancy. Ductography, however, is a painful procedure and has considerable limitations in detecting lesions that do not completely obstruct the ductal lumen, and also in detecting multiple lesions in the same duct [20]. Therefore the procedure is no longer widely practised in the management of PND.

### Ultrasonography

When the physical examination reveals a palpable abnormality in a localized area of the breast, ultrasonography may be useful in determining its characteristics; whether it is a cyst, an intracystic growth or a solid tumour. Modern high-resolution ultrasound techniques with 3-dimensional views [16] are helpful in visualizing intraductal disorders and are becoming a good complementary approach if not an alternative to traditional radiology techniques [16]. Furthermore, there is an increasing evidence that ultrasound-guided percutaneous stereotactic vacuum core biopsy is a reliable minimally invasive diagnostic and therapeutic modality in some clinical scenarios [21].

### MRI

Although magnetic resonance imaging (MRI) seems to be superior to both mammography and ultrasound for screening young women who are at high risk of developing breast cancer, its role in the management of papillomas is currently limited [22-24]. Intraductal papillomas present with a variable appearance on MRI ranging from occult to "small luminal mass" papillomas to irregular rapidly enhancing lesions that cannot be reliably distinguished from invasive malignancy [23]. In view of the high sensitivity of MRI, the absence of enhancement typical of malignancy in women with papillomas can be reassuring and supportive of conservative management [24]. However in clinical practice, the high cost, limited expertise and sub-optimal specificity of MRI remain obstacles to its widespread use [25].

More recently, MR-galactography has been shown to be of diagnostic value [16].

### Cytological examination of nipple discharge

Nipple discharge obtained by squeezing the nipple should be examined by means of papanicolaou or May-Giemsa staining in all patients. Cytology smears of the discharge material provide information about normality, atypia and malignancy and also about papillary formation of the exfoliated cells. Tests such as Hemoccult help to detect occult blood in the secreted fluid. Modern immunological tests can be performed on cytology smears where occurrence of high levels of carcinoembryonic antigen could indicate a latent malignancy [15]. Intraductal papillomas are characterised by the presence of tightly connected ductal cell clumps. The cells and nuclei are uniform in size, and non-mitotic. Erythrocytes are much more frequently seen in patients with intraductal papillomata. Some papillomas may have cytological appearances that are indistinguishable from those of ductal carcinoma in situ [26]. In lesions with associated atypia, however, the cytomorphologic features may overlap with those of low-grade carcinoma, and thus tissue biopsy is required for a definitive diagnosis [27].

### Mammary ductoscopy (MD)

Mammary ductoscopy (MD) is a new endoscopic technique that has been evolving over the last 15 years [20,28-30]. The initial scopes were of a large calibre with limited optics and lacked any working channels thus leading to a poor image quality. Since then, scopes have undergone a remarkable evolution. The new sub-millimetre fiberoptic micro-endoscopes measure between 0.7 and 1.2 mm in external diameter. Recently, Jacobs *et al *described new mini-endoscope with 0.55 mm external diameter [31]. Inserted through the ductal opening on the nipple surface, they allow direct visualization of the mammary ductal epithelium.

These scopes also provide working channels for insufflation, irrigation, ductal lavage, and possible therapeutic intervention [20]. The sharp clear magnified images viewed on a video monitor combined with the use of intraductal biopsy devices including micro-brushes and other biopsy tools allow the retrieval of tissue specimens under direct visualisation for cyto-pathological analysis [20,32,33]. Breast ductoscopy can be performed under local anaesthesia in the office setting with minimal discomfort and no reported complications.

MD offers the advantages of accurate localisation of pathology, ductal lavage under direct visualization, and intra-operative guidance especially for lesions deep within the ductal system [34]. In addition to visualising intraductal lesions, cytological analysis of endoscopically retrieved ductal lavage has been recently reported to be more accurate than simple discharge cytology [16,28,35]. In a cohort of 415 patients with PND, ductoscopy was successful in visualizing an intraductal lesion in 166 patients (40%). In these cases, ductal lavage following ductoscopy increased the yield of cytologically interpretable ductal epithelial cells 100-fold compared to discharge fluid alone [35]. The authors also reported that of the 11 cases of DCIS that were initially detected with a combination of MD and ductal lavage cytology, six were completely negative on clinical examination and mammography.

Despite these potential benefits of MD in the management of papillomas, this new technique is still not widely used in clinical practice due to the high cost and limited expertise. This position is likely to change in the near future.

## Treatment

Deciding on the appropriate surgery for intraductal papilloma is problematic due to the difficulty in discriminating between intraductal papilloma and breast cancer. Moreover, the significance of intraductal papilloma as a pre-cancerous lesion is controversial to surgeons. Patients with multiple papillomas do have an increased risk of developing breast cancer and should be kept under annual review with regular digital mammography if treated conservatively. In view of the high sensitivity of MRI, the absence of enhancement typical of malignancy in women with multiple papillomas can be reassuring and supportive of conservative management [24]. Because the risk is small, long term and affecting both breasts, long-term follow-up is more appropriate than prophylactic mastectomy. Patients who prove to have a solitary duct papilloma have insufficient increase in the risk of subsequent malignancy to justify routine follow-up.

From a historical perspective the philosophy of management of serous or sero-sanguineous nipple discharge changed radically in the last 50 years. Opinion regarding the likelihood of it being due to cancer was sharply divided early in the last century. Judd, [36] in 1917, reported a 57% incidence of cancer in 100 cases at the Mayo Clinic. In 1930, Adair reported 108 cases from the Memorial Hospital, with 47% malignant. In 1931, Cheatle and Cutler [37] argued strongly from pathological evidence that benign papillomas could progress to papillary carcinoma. This led to the simple mastectomy being the standard treatment for blood-stained discharge in many clinics.

However, in the last 30 years, a more conservative approach has become accepted, resulting particularly from the studies of Haagensen in the USA and Atkins and Wolff [38] in the UK, who all recognized that those patients whose discharge was due to intraductal papilloma were cured by removing the papilloma. Atkins developed the operation of microdochectomy and Haagensen [8] used a procedure that is intermediate between the microdochectomy of Atkins and the major duct excision operation of Urban [39]. If the discharge can be localized to a single duct, microdochectomy gives satisfactory results in younger patients with a minimal interference with the breast. In older patients where breast-feeding is not required, major duct excision may be preferable irrespective of whether the discharge is localized to one duct, both to avoid inconvenience of further discharge from a different duct and to provide a more comprehensive histology.

**Figure 1:**
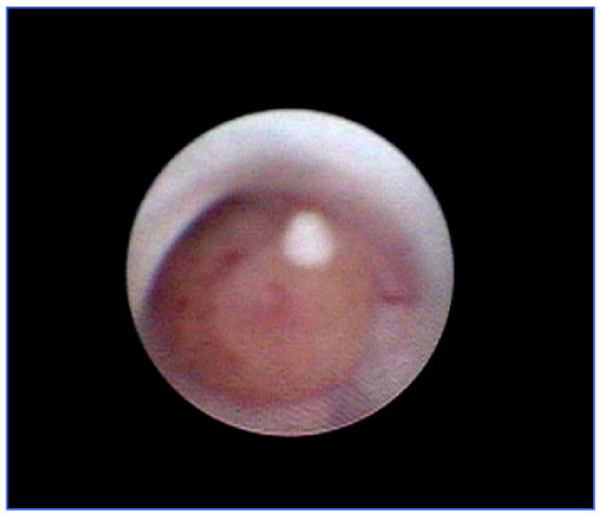
Mammary ductoscopy image showing intraductal papilloma.

MD has recently revolutionised the management of PND and breast papillomas. The only reliable way to both establish the diagnosis and control the discharge is ductal excision, the success of which is dependant on identifying the correct origin of the discharge. When a specific duct cannot be identified then blind excision of the retro-areolar ductal system is usually performed. MD offers the advantages of accurate localisation of pathology, ductal lavage under direct visualization (figure 1), and intra-operative guidance especially for lesions deep within the ductal system [20,30]. Furthermore, MD can detect multiple lesions within the same duct. The technique has the potential to reduce the number of duct excision procedures and minimise the extent of surgical resection. It is important to note that in some clinical scenarios, MD-guided intraductal biopsy can be diagnostic and curative and the discharge can resolve following the procedure [16]. This potential therapeutic role of MD-guided intraductal biopsy requires further evaluation and validation.

**Figure 2:**
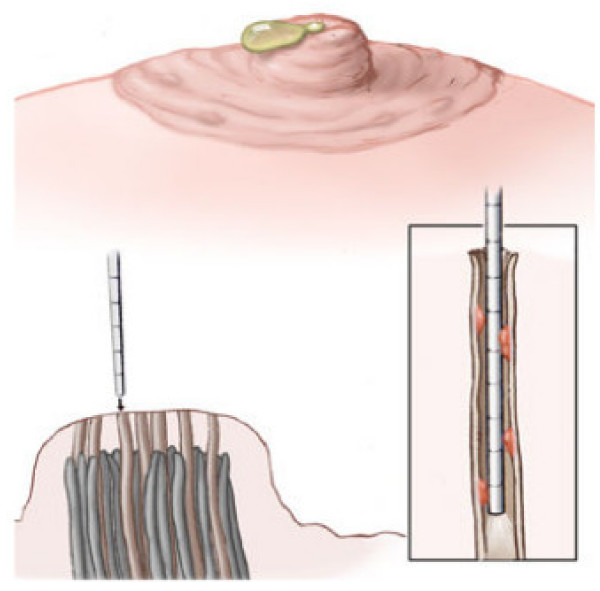
PND is identified, duct is dilated using appropriate lacrimal dilators, and MD is gently advanced into the ductal system until papilloma is identified. Blue dye is injected into the affected duct.

**Figure 3:**
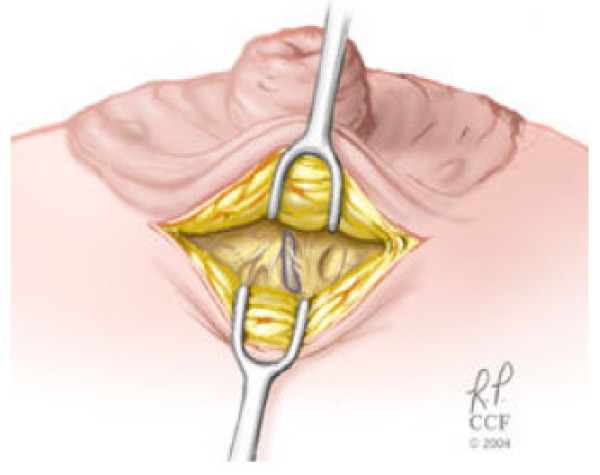
The duct is exposed by a skin incision inside the edge of the nipple-areola complex. The nipple is reflected away from the breast tissue in the area covered by the areola. The "blue dye" – involved duct is identified and divided as it enters the undersurface of the nipple

**Figure 4:**
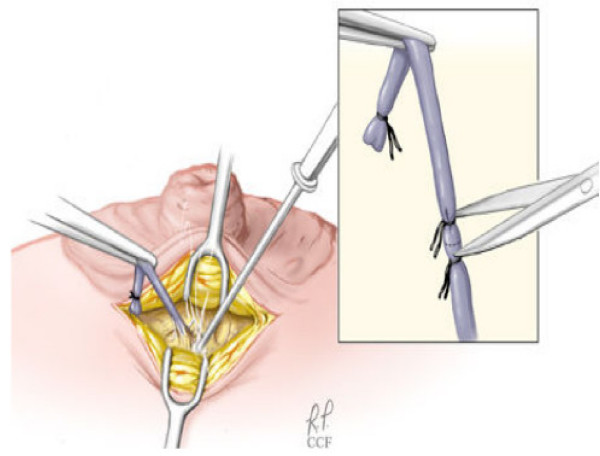
The dissection is carefully carried out caudally around the involved duct using the bovie cautery. During dissection, many dilated major ducts containing fluid can be identified. The nipple is gently everted, the subcuticular tissue approximated with an interrupted layer of 5-0 Vicryl suture. The skin is closed with a fine layer of 6-0-monocryl suture, and the wound is dressed with sterile strips.

Simple light trans-illumination through the skin during MD can be used to guide duct excision. We have recently described a new technique involving ductoscopy and the use of a blue dye to perform microdochectomy [40]. The procedure is performed in an outpatient surgical setting, under conscious sedation monitored control anaesthesia. Figures (2, 3, and 4) illustrate the operative technique used in MD guided microdochectomy. MD can also potentially reduce the need to perform duct excision in patients with PND due to benign disease thus resulting in cost savings.

Dietz et al [4] reported that duct cannulation was achieved in 105 (88%) of 119 patients with PND and ductoscopy directed duct excision could be performed in 104 (87%) of 119 cases. These authors also found that MD was more accurate than preoperative ductography (90% vs. 76%) in localising the relevant pathology. The pathological diagnoses were malignancy in 5 patients, papilloma in 84 cases, and hyperplasia in 16. In 22 patients, ductoscopy visualised multiple lesions or abnormalities beyond 4 cm. Such lesions would have been missed by blind duct excision.

Nevertheless, MD has recognised limitations. MD examines 1 – 2 ducts per breast and leaves the remaining 13 – 18 ducts that open at or just below the nipple surface unexamined. MD is also incapable of reaching the peripheral small branches of the ducts due to the scopes' diameter [20,30]. Thus it is unable to visualise the terminal duct-lobular unit (TDLU) where malignant lesions often originate. Furthermore complete occlusion of the duct by the lesion limits the role of MD in the management of PND.

**Figure 5:**
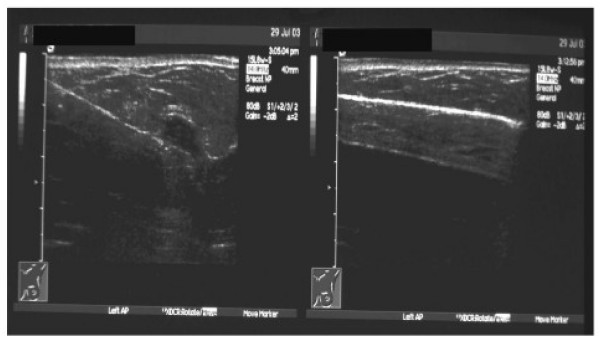
US-guided vacuum-assisted core biopsy of a mass lesion in the left breast (left). The lesion has resolved at the end of procedure (right).

If the papilloma is detectable on mammography and/or ultrasonography, then imaging-guided vacuum-assisted biopsy can be both diagnostic and curative (Figure 5).

Dennis et al evaluated the role of imaging-guided vacuum-assisted core biopsy as a minimally invasive method of obtaining a satisfactory diagnosis and eliminating the bothersome symptoms in patients presenting with nipple discharge. This study concluded that this technique allows safe and accurate tissue analysis and a high probability of terminating the symptomatic nipple discharge [41].

Finally if the discharge is not troublesome and all investigations show no evidence of malignancy, then the discharge can be managed conservatively with no need for surgical intervention.

**Figure 6:**
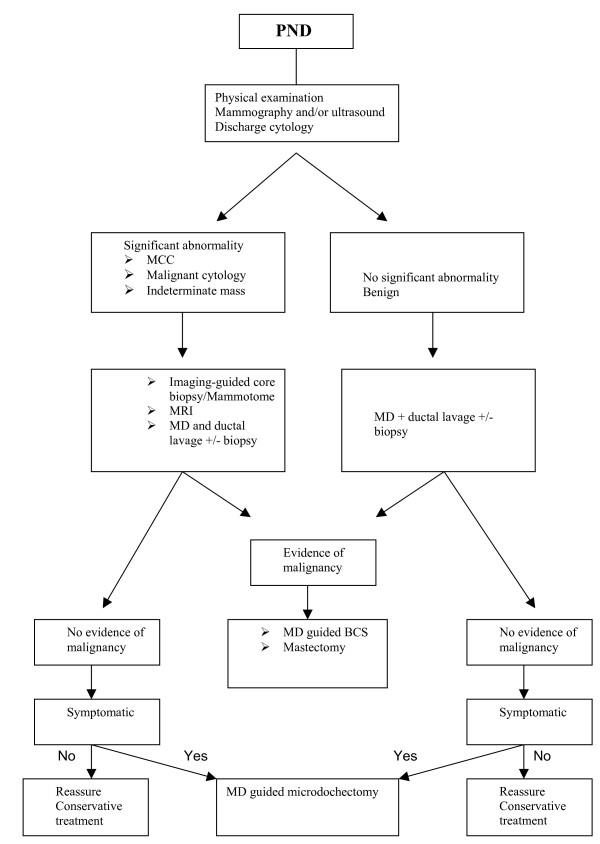
A proposed algorithm for the management of PND. PND: pathological nipple discharge. MCC: Micro calcifications. MD: Mammary Ductoscopy. BCS: Breast conserving surgery

Figure 6 shows a new proposed algorithm for the treatment of papillomas.

## Future prospects

Current biopsy tools used with MD need further evaluation and validation, as the challenge of verifying endoscopic appearances with a histological diagnosis has not yet been overcome. The future development of reliable intraductal biopsy tools capable of obtaining tissue sample under direct visualisation sufficient for histological diagnosis can enhance further the role of MD in the management of PND and papillomas. The potential therapeutic role of MD-guided intraductal biopsy [16] requires further evaluation. MD can also complement other novel screening modalities such as molecular profiling including proteomics in differentiating benign papillomas from pre-malignant and malignant lesions [42].

Moreover, the addition of radiofrequency [43] to MD as a curative endoscopic procedure is worthy of investigation. These potential applications underscore the need to develop reliable methodology to mark the ducts harbouring pathology.

## List of abbreviations

CAD: Computer-aided detection

DCIS: Ductal Carcinoma In-situ

JP: Juvenile Papillomatosis

MCC: Micro-calcifications

MD: Mammary Ductoscopy

MRI: Magnetic Resonance Imaging

PND: Pathological Nipple Discharge
